# Correction

**DOI:** 10.1093/eurpub/ckae174

**Published:** 2024-11-12

This is a correction to: Petra Ožbolt, Andrea Backović Juričan, Janja Križman Miklavčič, S02-3: Slovenian Approach to Enhance Physical Activity Among Vulnerable Populations, *European Journal of Public Health*, Volume 34, Issue Supplement_2, September 2024, ckae114.204, https://doi.org/10.1093/eurpub/ckae114.204

and

Gyöngyvér Lacza, Réka Veress, S02-4: How does the Hungarian Active Lifestyle Strategy address social inclusion?, *European Journal of Public Health*, Volume 34, Issue Supplement_2, September 2024, ckae114.205, https://doi.org/10.1093/eurpub/ckae114.205

In the originally published versions of these two abstracts, the lists of presenters were erroneously transposed. The correct names are now attached for the presenters of each abstract respectively.

